# EVALUATING EXPENDITURES AND UTILIZATION OF OHIO'S INTEGRATED MEDICARE AND MEDICAID PROGRAM

**DOI:** 10.1093/geroni/igac059.747

**Published:** 2022-12-20

**Authors:** John Bowblis, Robert Applebaum, Matt Nelson

**Affiliations:** Miami University, Oxford, Ohio, United States; Miami University, Oxford, Ohio, United States; Miami University, Oxford, Ohio, United States

## Abstract

In 2014, 29 of 88 Ohio counties implemented MyCare, which integrated Medicare and Medicaid for dually eligible Ohioans. Using an intent-to-treat, difference-in-difference framework we examined medical expenditures and utilization associated with the implementation of MyCare. Specifically, we compared dually eligible Ohioans in MyCare counties to those in non-MyCare counties from 2012 to 2018. Overall medical expenditures were lower in the MyCare counties post implementation compared to non-Mycare counties, with most of the difference attributed to Medicaid. The effects were larger for individuals in the community compared to long-term services and supports (LTSS) users. The implementation of MyCare is associated with a decrease in the use of nursing homes, a large increase in hospice, and among LTSS users not in a nursing home decreases in the utilization of home and community-based services. Interestingly, the proportion of individuals in MyCare counties classified as an LTSS user increased after the implementation of MyCare.

